# Flow-Mediated Factors in the Pathogenesis of Hypoplastic Left Heart Syndrome

**DOI:** 10.3390/jcdd9050154

**Published:** 2022-05-12

**Authors:** Anum Rahman, Rajiv R. Chaturvedi, John G. Sled

**Affiliations:** 1 Mouse Imaging Centre, The Hospital for Sick Children, Toronto, ON M5T 3H7, Canada; john.sled@utoronto.ca; 2 Translational Medicine, The Hospital for Sick Children, Toronto, ON M5G 1X8, Canada; 3 Department of Medical Biophysics, University of Toronto, Toronto, ON M5G 1L7, Canada; 4 Division of Cardiology, Department of Pediatrics, The Hospital for Sick Children, Toronto, ON M5G 1X8, Canada; rajiv.chaturvedi@sickkids.ca

**Keywords:** hypoplastic left heart syndrome, mouse, fetus, blood flow, mitral valve, aorta, stenosis, left ventricle, fetal aortic valvuloplasty, hemodynamics

## Abstract

Hypoplastic left heart syndrome (HLHS) is a life-threatening congenital heart disease that is characterized by severe underdevelopment of left heart structures. Currently, there is no cure, and affected individuals require surgical palliation or cardiac transplantation to survive. Despite these resource-intensive measures, only about half of individuals reach adulthood, often with significant comorbidities such as liver disease and neurodevelopmental disorders. A major barrier in developing effective treatments is that the etiology of HLHS is largely unknown. Here, we discuss how intracardiac blood flow disturbances are an important causal factor in the pathogenesis of impaired left heart growth. Specifically, we highlight results from a recently developed mouse model in which surgically reducing blood flow through the mitral valve after cardiogenesis led to the development of HLHS. In addition, we discuss the role of interventional procedures that are based on improving blood flow through the left heart, such as fetal aortic valvuloplasty. Lastly, using the surgically-induced mouse model, we suggest investigations that can be undertaken to identify the currently unknown biological pathways in left heart growth failure and their associated therapeutic targets.

## 1. Introduction

Hypoplastic left heart syndrome (HLHS) is a severe form of congenital heart disease that develops in utero and is marked by the failure of left heart structures to grow appropriately. Clinically, HLHS is defined by the presence of a non-apex-forming left ventricle, mitral and aortic valve stenosis or atresia, and hypoplasia of the aorta. In contrast, the ventricular septum in HLHS is typically intact with concordant atrioventricular and ventriculoarterial connections [1,2,3]. These lesions result in obstruction of blood flow from the left outflow tract of the fetal heart that, when severe, is characterized by an absence of antegrade blood flow through the aortic valve and retrograde flow through the aortic arch and ascending aorta originating from the arterial duct. Postnatal physiological closure of the arterial duct is fatal in affected individuals without intervention.

Although the term hypoplastic left heart syndrome was first used by Drs. Noonan and Nadas in the 1950s [4], the etiology of this syndrome remains to be elucidated. This knowledge gap poses significant challenges for developing treatments that can slow or reverse the progression of HLHS. Indeed, management of HLHS is largely limited to cardiac transplantation or a three-stage palliative surgical reconstruction procedure that is performed in early childhood [5]. These procedures are associated with significant morbidity (heart failure, arrhythmia, and hepatic dysfunction) [5], mortality (10-year survival rate ranges between approximately 40–55%) [6,7,8], and healthcare resources (median inpatient charges of approximately USD 376,403/child for surgical palliation and USD 582,920/child for cardiac transplantation) [9].

There is an urgent need to establish the underlying mechanisms of HLHS and identify new therapeutic targets. In this review, evidence is presented that supports the role of blood flow in the pathogenesis of HLHS and the implications for the treatment of HLHS.

## 2. The Flow Theory of Hypoplastic Left Heart Syndrome

Several lines of evidence suggest that decreased blood flow through the left heart is important in the pathogenesis of HLHS. First, routine ultrasonographic studies performed between 18–22 weeks of gestation (or, more recently, at 11 weeks [10]) have shown that, in some cases, an initially identified aortic stenosis (but a normal-sized or dilated left ventricle) can progress to HLHS in later gestation [11,12,13,14,15]. Second, in chick embryo [16,17,18] and fetal lamb models [19,20], the surgical reduction of inflow through the mitral valves has resulted in several features associated with HLHS, such as a hypoplastic left ventricle and ascending aorta. Third, it has been suggested that the anatomy of the atrial septum may contribute to left heart hypoplasia. For example, in 6–22% of HLHS cases [21,22], the atrial septum is restrictive or closed. This may be a primary defect of atrial anatomy or secondary to left atrial hypertension closing the septal flap [14,23,24]. In either case, right-to-left atrial shunting decreases and may reverse direction, accompanied by a fall in left heart blood flow, contributing to hypoplasia of the left heart. Lastly, a recent study of induced pluripotent stem-cell-derived cardiomyocytes (iPSC-CMs) obtained from an HLHS-affected family trio showed that in a proband with an *MYH6* variant, iPSC-CMs had decreased shortening, slower contractions, and dysmorphic sarcomeres. This latter phenotype was also observed in the atrial but not in the ventricular tissue collected from HLHS patients, suggesting that intrinsic defects in atrial contractility might lead to decreased inflow into the left heart and, consequently, a hypoplastic left ventricle [25].

While these clinical findings and animal studies support the idea that decreased blood flow can lead to decreased growth of left heart structures, the history and pathogenesis of HLHS in utero is still unknown. This has made it challenging to determine in which clinical presentations an initially identified at-risk left ventricle will progress to severe hypoplasia or recover and be amenable to biventricular physiology with intervention [26,27,28,29]. Progress on understanding the pathogenesis of HLHS has been hampered in part by the limitations of available animals that do not survive to term or unsatisfactorily recapitulate the anatomical features of HLHS. Furthermore, although HLHS can be reliably detected by 18–22 weeks of gestation with ultrasound [30], it is not known when in utero this disease first develops.

Recently, our group attempted to answer these questions through the creation of the first mouse model of isolated HLHS [31]. Our approach to creating this model was guided by two observations: (i) the HLHS heart in utero displays an intact ventricular septum, normally aligned great vessels, and concordant atrioventricular connections; and (ii) that this contrasts with the chick embryo model, in which left atrial ligation is performed during cardiogenesis and structural abnormalities other than left heart hypoplasia, such as ventricular septal defects, can occur (in approximately a quarter of surgically manipulated embryos) [18,32]. Therefore, we hypothesized that the insult leading to left heart growth failure likely occurs in the fetal period after the early cardiac developmental events have occurred (i.e., after looping, convergence, wedging, and septation events have taken place by weeks 8–9 of gestation [33,34]).

**Figure 1:**
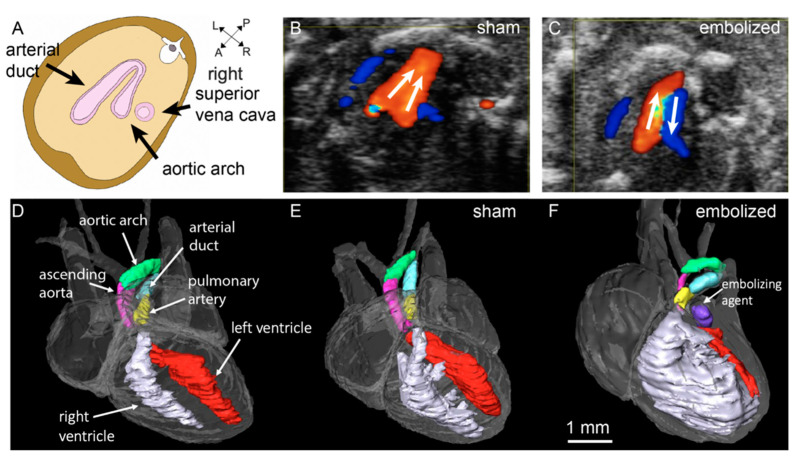
Aortic arch blood flow and left heart structural abnormalities observed at gestational term in hypoplastic left heart syndrome (HLHS) mice. Referring to the anatomical reference (**A**), blood flow through the aortic arch was antegrade in the sham (**B**) (needle advancement into the left atrium but without any embolization) and retrograde in the embolized fetus (**C**), as seen in color-flow Doppler ultrasound. High-resolution three-dimensional magnetic resonance imaging (MRI) of the fetal heart (**D**) in which the major heart structures are manually colored. Comparing a sham (**E**) and embolized fetus (**F**), the left ventricle was non-apex-forming, and the chamber volume was greatly reduced by embolization of the left atrium. The caliber of the ascending aorta vessel was also smaller compared to the pulmonary trunk in the embolized fetus. L: left; R: right; A: anterior; P: posterior. Figure modified from [31] under a CC-BY 4.0 license.

To test this hypothesis in a mouse model, we needed to establish the age equivalent of 8–9 weeks of gestation in the human heart. Previous work by Krishnan et al. [35] had established that a mouse heart resembles its adult configuration by embryonic day (E) 14.5, and we verified this by dissection and magnetic resonance imaging (MRI) [31]. We created a model of HLHS by microinjecting the left atrium cavity in fetal mice at E 14.5 with an embolizing agent under high-frequency ultrasound guidance. Our goal was to block blood flow into the left ventricle through the mitral valve. At E 18.5 (term), we observed key features of HLHS on ultrasound and MRI, including retrograde aortic arch flow, non-apex-forming left ventricles, hypoplastic aortic valves, and ascending aortas (Figure 1). Furthermore, all fetal mice exhibited normally aligned great vessels and an intact ventricular septum. Our results thus give support to the “no-flow, no-grow” hypothesis of HLHS and indicate that (i) decreased blood flow through the left heart can lead to growth failure and (ii) a subset of HLHS cases likely occur in the fetal period post-cardiogenesis, rather than in the embryonic period.

In the clinical setting, HLHS has been detected as early as 12 weeks of gestation with ultrasound [36]. This is still 3–4 weeks after the completion of cardiogenesis [33,34] during a period when the heart is growing rapidly. The left atrium embolization model demonstrates that HLHS can occur after cardiogenesis, but does not exclude it from occurring or being initiated earlier. It may be feasible to perturb blood flow in embryonic mice during cardiogenesis (i.e., prior to E 14.5) by left atrial embolization to address this.

## 3. Flow-Mediated Mechanisms of Left Heart Development

In normal development, the fetal period represents a key window of susceptibility to left heart hypoplasia, as this period is marked by substantial increases in cardiac chamber dimensions and elongation and thinning of the valve leaflets [35]. In terms of valvular development, it has been found that elongation and thinning of the valves can be independently regulated by fluid shear stresses, tensile stresses, and chronic cyclical stretches through mechanotransduction programs [37,38,39]. For instance, as reviewed elsewhere [40,41], it has been suggested that the side of the semilunar valve that faces the aorta (arterial side) experiences oscillatory shear stress (in response to reversing flows), while the side that faces the ventricle experiences laminar shear stress (in response to unidirectional flow). Recently, using a combination of in vivo and in vitro assays in fetal mice and chick embryos, it was demonstrated that, on the arterial side of the valve, mechanotransduction of low oscillatory shear stress through canonical Wnt signaling resulted in increased endocardial cell BMP signaling and cellular proliferation, leading to elongation and growth of the semilunar valve on the arterial side [37]. Conversely, mechanotransduction of high laminar shear stress on the ventricular-facing side of the semilunar valve through Notch1 signaling led to decreased Wnt/BMP activity and decreased endocardial cell proliferation [37]. These results suggest that differential shear stresses experienced by aortic valve leaflets can be transduced through mechanosensitive signaling pathways to influence cellular proliferation in a regional-specific manner, thereby promoting semilunar valve elongation and remodeling in fetal life. Perturbations in shear stress patterns may, therefore, lead to the development of the thickened stenotic and atretic valves observed in HLHS.

In addition to the effect of mechanical forces on fetal aortic valve development, there is also evidence of these forces being important in atrioventricular valve development. Recent studies have shown that tensile stress and cyclical stretch alone can modulate elongation and thinning of the atrioventricular valves [38,39]. In the chick embryo model of left heart hypoplasia, partial ligation of the left atrium (which impairs cyclical loading) led to inhibition of the mechanosensitive Rac1 GTPase protein, and this was associated with abnormal mitral valve remodeling (i.e., underdeveloped mitral valves) [39]. In vitro assays confirmed that, during normal atrioventricular valve development, chronic cyclical stretch led to a relative increase in Rac1 activation, and this was associated with extracellular matrix compaction and cellular elongation [39], processes that are necessary for the thinning and maturation of mitral valve leaflets with increasing gestation. In terms of left ventricle hypoplasia, decreased blood flow in the chick embryo model was associated with decreased wall shear stress and myocyte cell proliferation, as well as subendocardial fibrosis and hypoxia [17,42,43,44]. These findings are similar to those observed by Gaber et al. in postmortem human specimens, where the HLHS left ventricle myocardium showed increased hypoxia-inducible factor-1 alpha expression (suggesting hypoxia), increased cellular senescence and fibrosis, and a decreased number of cardiomyocyte cells [45]. Although not causative, these findings suggest that altered blood flow in the left heart may lead to ventricular hypoplasia and endocardial injury, possibly due to changes in mechanical forces and tissue hypoxia.

Taken together, emerging evidence suggests that the mechanical forces imparted by blood flow and oxygenation content can independently mediate growth and remodeling of left heart structures through mechanosensitive and hypoxia-sensing programs. Whether these programs are altered in HLHS and whether they are responsible for left heart growth failure and endocardial fibroelastosis remains to be determined. The recent creation of a fetal mouse model that survives to gestational term [31] provides a novel opportunity to map the natural history of HLHS by tracking how the mechanical forces evolve in utero and identifying the cellular and genetic mechanisms responsible for left heart growth failure. 

## 4. Surgical Interventions to Treat Hypoplastic Left Heart Syndrome

The “no-flow, no-grow” hypothesis of HLHS has led to the adoption of fetal aortic valvuloplasty by several clinical centers [46]. This procedure is performed at mid-gestation with the goal of relieving fetal aortic stenosis and preventing progression to HLHS. Current era results from the two largest-volume centers show procedural success of 94–96% and biventricular circulation in 55–59% of liveborns after fetal aortic valvuloplasty [26,47]. However, biventricular circulation after a recent-era fetal aortic valvuloplasty has a probability of transplant-free survival to 6 years (0.82; 95% confidence interval 0.73–0.89) similar to that for patients managed as univentricular hearts (0.72; 95% confidence interval 0.61–0.82) in cases of HLHS due to aortic stenosis and mitral stenosis as reported in the Single Ventricle Reconstruction trial [48].

The modest effect of these interventions on long-term biventricular circulation may reflect suboptimal timing or failure to address all aspects of impaired heart function. Indeed, the optimal timing for clinical interventions to rescue the heart is currently not known. This is an important consideration, as studies in mice suggest that ventricular cardiomyocyte cellular proliferation decreases steadily in utero. For instance, while the thymidine labeling index of cardiomyocytes (a marker for proliferation) is approximately 23% at E 14 and 20% at E 16, it decreases to about 10% near gestational term [49,50]. Corresponding to these findings, the number of cardiomyocyte nuclei per unit area increases between E 14–E 16 and then decreases thereafter, suggesting that, while cardiac chamber growth is primarily due to hyperplasia (proliferation) in the early post-cardiogenesis period, it begins to switch to hypertrophic growth (cell enlargement) in later gestation [49].

In the fetal mouse model of HLHS, we observed that embolization at E14.5 resulted in HLHS by E 18.5 [31]. Although we did not examine intermediate timepoints, we speculate that left ventricular hypoplasia is likely present by E 16.5 in our mouse model, given that an earlier study [49] suggested that the switch between proliferative and hypertrophic growth begins to occur around E 16 in mice. In terms of human fetal heart development, studies investigating the transition between ventricular hyperplasia and hypertrophy are lacking. However, it is possible that interventions to improve blood flow and rescue the left ventricle after this switch occurs (approximately E 16 in mice) may not be as beneficial. Furthermore, compared to human pregnancies, in which the fetal period of cardiac development extends between approximately weeks 8–9 of gestation and birth, the testing of novel prenatal interventions in the fetal mouse model of HLHS will have to occur during a narrower window of development, between the completion of cardiogenesis and birth (between E 14.5–E 18.5).

In addition to these considerations of timing, it will be important for proposed interventions to account for the extent that perturbed flow is responsible for the anatomical heterogeneity observed in HLHS. For instance, a postmortem examination of 78 HLHS human specimens has revealed three separate subgroups based on the left ventricle phenotype [51]. In all cases, the left ventricle was non-apex-forming, but the dimensions of the ventricle chamber and wall thickness varied. The three subgroups were defined as follows: a slit-like ventricular cavity, a miniaturized ventricular cavity, and a small, thick-walled left ventricular cavity with endocardial fibroelastosis. Currently, the mechanisms driving these ventricular subtypes are not known, and the observed overlap in the aortic and mitral valve morphologies between the different ventricular subtypes suggests that factors other than impaired blood flow may be important.

**Figure 2:**
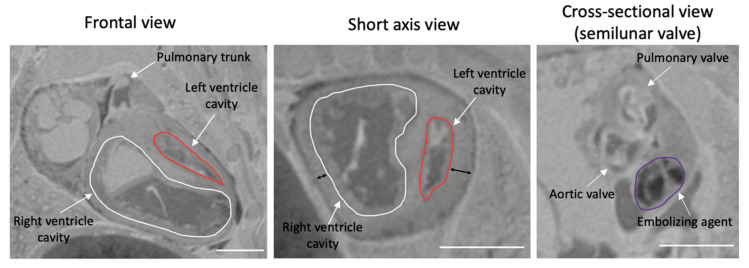
Slices from a three-dimensional magnetic resonance image of a gestational-term CD-1 mouse fetal heart. In the frontal view, a hypoplastic left ventricle cavity can be observed, and it is non-apex-forming. In the short-axis view (mid-ventricular level), myocardial wall thickness measurement was performed on the two-dimensional MR slice at the locations depicted by the double-headed black arrows. The left ventricle free-wall myocardium was thicker (0.27 mm) compared to the right ventricle free-wall myocardium (0.13 mm). In the cross-sectional view, the aortic valve annulus was comparable in size to the pulmonary valve. Scale bar: 1 mm.

Thus, reproducing these phenotypes in animal models may offer insight into the mechanisms that drive HLHS in individual patients. In the fetal mouse model of HLHS [31], for instance, reduced blood flow through the mitral valves at E 14.5 resulted in a non-apex-forming left ventricle, often with a slit-like ventricular cavity (Figure 1). In a more recent study by our group (unpublished), we performed the left atrium embolization procedure at E 14.5 in a larger cohort and observed on MRI at gestational term an instance where the left ventricle morphology was similar to the thick-walled ventricle phenotype (Figure 2). Surprisingly, the fetal mouse with the thick-walled hypoplastic left ventricle had a normal-sized aortic valve and ascending aorta. In the past, it has been suggested that left ventricle hypoplasia without any significant aortic hypoplasia gives weight to the hypothesis that intrinsic defects are the primary drivers of left ventricular growth failure in HLHS [52,53,54]. This is because abnormal hemodynamics would be expected to affect both the left ventricle and the aorta.

Although the results presented here are preliminary—we did not characterize endocardial fibroelastosis or valvular morphology in this mouse—they suggest that reduced blood flow in the left heart can result in varying left ventricle and aortic phenotypes within the HLHS spectrum. This might be due to genetic variation between subject mice (i.e., CD-1 mice, as used in this study, are an outbred strain). A histological investigation of valvular morphology, endocardial fibroelastosis, and hemodynamic forces in utero, along with capturing the genetic heterogeneity between mice, may help clarify the role of impaired flow and intrinsic genetic factors in the development of HLHS subtypes. This will be important for determining why some patients are at risk for developing HLHS, as well as in which patients surgical interventions or emerging therapies such as maternal hyperoxygenation [55,56,57,58] (which has been associated with improved left heart preload and growth in select cases) would be the most beneficial.

## 5. What Causes Flow Disturbances in Hypoplastic Left Heart Syndrome?

Although evidence for the “no flow, no grow” hypothesis of HLHS is accumulating, the cause of the initial insult(s) that leads to perturbation in blood flow is often not known. Recently, iPSC studies have shown the presence of intrinsic defects in the myogenic and endocardial programs of HLHS left ventricle tissue [59,60,61]. Findings from these studies have included differentiation deficiencies among the first heart field cardiac progenitor cells, premature cell-cycle exit, impaired cardiomyocyte proliferation and maturation, increased cardiomyocyte apoptosis, abnormalities in excitation–contraction coupling, and endocardial defects. Thus, it is possible that, in HLHS, intrinsic defects are already present prior to the presence of hemodynamically significant events.

**Figure 3:**
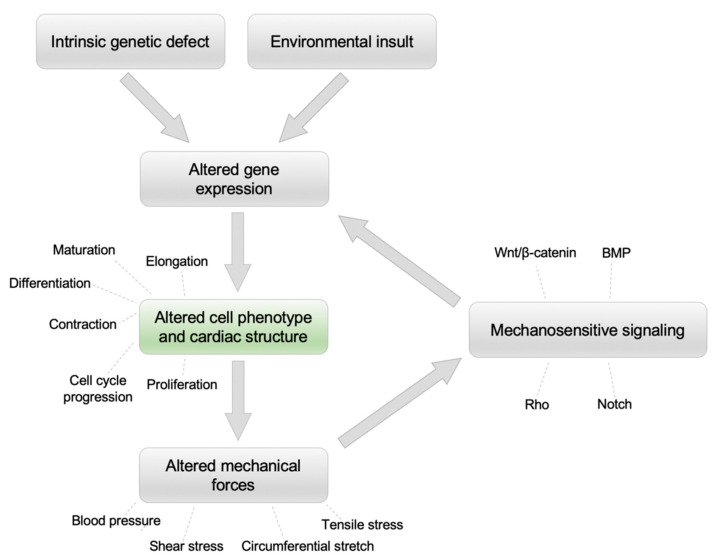
Schematic of how upstream factors (intrinsic genetic disturbances or environmental insult) can converge on downstream processes to cause hypoplastic left heart syndrome (HLHS).

While these findings are important, it is yet unknown how intrinsic disturbances in cardiac development and cell cycle programs of the left heart ultimately cause HLHS, although it is likely that hemodynamics play an important role. Indeed, any disturbance (genetic or otherwise) that alters heart development often results in perturbations to blood flow [62,63]. Thus, it is possible that initial genetic disturbances in the left heart valvular or ventricular primordial tissue leads to changes in cell numbers, shape, and contractility, which then alters the hemodynamically driven mechanical forces acting on the heart. In response to altered hemodynamics, mechanosensitive signaling pathways may further cause changes in underlying genetic programs and cell properties. Together, downstream cellular changes stemming from intrinsic defects and abnormal blood flow may then interact over the course of gestation to ultimately produce the clinical HLHS phenotype (Figure 3). In this regard, some genetic variants identified in whole-exome sequencing and iPSC studies of HLHS are involved in the Notch and Wnt signaling pathways, which are also known to be mechanosensitive [37]. Regardless of the initial genetic disturbances and consequent changes in cellular properties and hemodynamics, these processes likely combine in a specific manner (and time) so that the left heart becomes hypoplastic but the early morphological events in cardiac development (such as septation and wedging) are unaffected.

## 6. Conclusions and Future Directions

The findings from the surgically induced mouse model of HLHS shows that decreased blood through the left heart following the closure of interventricular communication can lead to the development of hypoplastic left heart syndrome [31]. This suggests that blood flow perturbations are an important causal factor in the pathogenesis of this disease and that a subset of cases may develop in the fetal rather than the early embryonic period.

Furthermore, since the mouse model described in this review reproduces the structural and circulatory abnormalities observed in fetal HLHS and survives to gestational term, it provides a unique opportunity to answer previously intractable questions, such as mapping the evolution of HLHS in utero. Specifically, by performing embolization earlier or later than E 14.5 and in different strains of mice, it may be possible to determine how the timing of blood flow perturbation and the genetic background interact to produce the ventricular and valvular heterogeneity observed within the HLHS spectrum. Second, post-cardiogenesis fetal left ventricular inflow obstruction resulting in HLHS has also been demonstrated in mid-gestation fetal lambs [20], and we anticipate that it can be demonstrated in all mammalian species and, potentially, in any four-chambered hearts with technical adaptations. We speculate that species in which it is feasible to block left ventricular inflow soon after cardiogenesis and that have a long gestation may demonstrate severe left heart hypoplasia. Lastly, by using this mouse model to investigate processes downstream of the initial genetic or environmental insult that are often unknown (Figure 3), it may be possible to identify the common molecular and cellular pathways that are abnormal in HLHS subtypes. This will be an important first step in identifying novel therapeutic targets that are urgently lacking in the treatment of HLHS.

## Data Availability

Not applicable.
